# Correction: Ultrasensitive Detection of Circulating LINE-1 ORF1p as a Specific Multicancer Biomarker

**DOI:** 10.1158/2159-8290.CD-24-0862

**Published:** 2024-09-03

In the original version of this article (1), the pie chart depicting ORF1p tissue expression in ovarian carcinoma (“ovary”) in Fig. 1D was omitted by the compositor during the production of the manuscript. Figure 1D has been corrected in the latest online HTML and PDF versions of the article. The publisher regrets the error.
